# Seroepidemiological Study of *Toxoplasma gondii* Infection among Psychiatric Patients in Mashhad, Northeast of Iran

**Published:** 2017

**Authors:** Ebrahim ABDOLLAHIAN, Reza SHAFIEI, Naghmeh MOKHBER, Kurosh KALANTAR, Abdolmajid FATA

**Affiliations:** 1. Psychiatry and Behavioral Sciences Research Center, Faculty of Medicine, Mashhad University of Medical Sciences, Mashhad, Iran; 2. Vector-Borne Diseases Research Center, North Khorasan University of Medical Sciences, Bojnurd, Iran; 3. Dept. of Parasitology and Mycology, School of Medicine, Mashhad University of Medical Sciences, Mashhad, Iran; 4. Dept. of Immunology, School of Medicine, Shiraz University of Medical Sciences, Shiraz, Iran; 5. Research Center for Skin Diseases and Cutaneous Leishmaniasis, Emam Reza Hospital, Mashhad University of Medical Sciences, Mashhad, Iran

**Keywords:** *Toxoplasma gondii*, Psychiatric patients, ELISA

## Abstract

**Background:**

Psychiatric patients have an increased risk of some infections like toxoplasmosis. Investigations on *Toxoplasma gondii* infection among psychiatric patients have been limited in Mashhad, Northeast of Iran. In this case-control study, prevalence of T. gondii was investigated by serological method.

**Methods:**

This case-control study was performed among psychiatric patients admitted to Avicenna Hospital in Mashhad, Northeast of Iran. Three hundred and fifty inpatients and 350 controls were examined in 2012–2013 for detection of IgG and IgM antibodies against *T. gondii* in their blood sera by ELISA. Socio-demographic and clinical manifestations of the patients were obtained.

**Results:**

Anti-*T. gondii* IgG antibodies was found in 164 (46.85%) of 350 psychiatric inpatients and 120 (34.28%) of 350 controls. Seventeen (4.85%) of psychiatric individuals and 3 (0.85%) of control group were IgM+/IgG− indicating acute form of toxoplasmosis. There were no statistically significant differences between the case and control groups. In patient group, schizophrenic patients had the highest positive rate (46.28%) and bipolar mood disorder had the second most prevalent rate (20%). Of 162 schizophrenia patients, 65 (40.1%) had latent infection which was higher than that observed in controls.

**Conclusion:**

The prevalence of *T. gondii* infection among psychiatric patients suffering from schizophrenia was more in Mashhad, compared with control group.

## Introduction

Psychiatric patients are in high risk of some infections, not only because of their life style; however, it could be a common etiologic process. The relationship between infectious diseases and psychiatric disorders has rooted in the epidemiologic surveys that reports high co-morbidity of these conditions (1–3). Some infections have been declared to play a role in the etiology of some major psychiatric problems (4, 5).

*Toxoplasma gondii,* is one of the obligate intracellular protozoan parasite in the phylum Apicomplexa with a worldwide distribution in a wide variety of intermediate hosts including humans and other mammals (6). Humans may become infected through with ingestion of oocysts in cat feces, or by eating meat of infected animals (7). Primary acute toxoplasmosis may be developed in each trimester of pregnancy and causes severe damage to the foetus (8, 9). Subsequently, usually acquired infections are asymptomatic but in some patients presented by ocular and central nervous system manifestations. *T. gondii* may affect dopamine levels into the brain, causing in alterations in CNS (10). Earlier investigations observed that latent *Toxoplasma* infection might affect behavior (11), perhaps being a contributory, or even causative, factor in some psychiatric disorders, including depression, anxiety and schizophrenia (1, 10, 12–14).

Several factors affect prevalence of toxoplasmosis including age, rural or urban setting, socioeconomic criteria and nutritional habit (15). Furthermore, seroprevalence of infection rates vary from 10% to 70% in Asia (16), 24% to 57.5% in two Iranian populations (17, 18). It is estimated to be about 50% in Iran; therefore, toxoplasmosis continues to be a public health problem in Iran (19)**.** In Northeast of Iran, there is no data about seropositive of *T. gondii* infection from health and patients suffer from psychiatric problems, and there is no information about risk factors between *T. gondii* antibodies and psychiatric disorders.

The aim of this investigation was to check the prevalence of antibodies against *T. gondii* in patients with psychiatric and mood disorders and in a matched group of control subjects.

## Materials and Methods

This case-control study was performed in 2013 between two populations: psychiatric/mood disorders patients and control group. Since Dec 2011 to Mar 2012, all patients referred to the only Avicenna Hospital in Mashhad, Northeast of Iran, were invited to enroll in this study. The patients had been diagnosed clinically by psychiatrics. All psychiatric patients were included in the study based on the following inclusion criteria: 1) psychiatric inpatients, 2) aged >16 yr, 3) consent to participate in the study. During the study period, 350 psychiatric disorders patients were hospitalized. The age range of the population was 16–75 (35±11.61) yr old. All patients had no family history of schizophrenia, no evidence of immunodeficiency or other immunologic abnormalities, no history of head trauma, previous meningitis/encephalitis and brain surgery.

### Sampling

Three hundred and fifty healthy volunteers were selected as control group. They were screened for the absence of physical and psychiatric disorders and matched to patients according to sex, socioeconomic status, and age (38±13.2 yr old), matched with study group (*P*>0.05).

The Research Ethical Committee of Mash-had University of Medical Sciences, Iran, approved this study. All participants signed informed consent form.

### Serological examination

A sample of 5 ml blood was collected from each psychiatric patients and control; then serum separated by centrifugation at 1000 r.p.m. and stored at −20 °C. All samples labeled by blind numbers unrecognized to other colleagues in this study. The levels of specific IgG and IgM antibodies to *T. gondii* in the serum samples were measured using a commercial enzyme immunoassay kit (Pishtaz Teb Diagnostics, Tehran, Iran). The IgG and IgM antibody titers were read at optical density (OD) of 490 nm using automatic ELISA reader (Spectra, Molecular Devices, USA). ELISA cut off for positive and negative results were 10IU/ml. The results below that considered as negative and upper than that considered as positive.

SPSS software ver. 16.0 was used for statistical analysis. The relative proportions were calculated with a confidence interval of 95%. Possible associations were identified using the Chi-Square and Fisher’s exact statistical tests at a significant level of 5%.

### Statistical analysis

Socio-demographic data including age, birthplace, residence, marital status, occupation, educational level and socio-economic level were obtained from all patients. Clinical data including blood transfusion or transplant history; and behavioral data including animal contacts, cat attender, consumption of meat (raw or undercooked lamb, beef, goat, chicken, sea food and bird), unpasteurized dairy products, contaminated water, improper washed raw vegetable or fruits, contact with soil (gardening or agriculture), were obtained. Clinical diagnosis was confirmed by means of the Structured Clinical Interview as mentioned in the Diagnostic and Statistical Manual of Mental Disorders, Fourth Edition (DSM-IV) criteria (20).

## Results

The number of male individuals were 263 (75.1%) and 170 (48.6%) in patient group, respectively. More than 74% of the patients were citizens of Mashhad. The anti *T. gondii* IgG+/IgM+ antibody was positive in 46.85% and 34.28% of both case and control group, respectively. The seroprevalence of latent *T. gondii* infection in the populations studied according to age groups and gender. As you can observe the prevalence of the infection in both patients and controls increased with age (Table 1).

**Table 1:** Seroprevalence of anti-*T. gondii* IgG/IgM antibodies in psychiatric patients and controls according to age groups and gender

**Age group (yr)**	**Controls**				**Psychiatric patients**			
	***T. gondii*** **infection**				***T. gondii*** **infection**			
	**IgG+ No. %**	**IgM+**	**No. tested**		**IgG+ No. %**	**IgM+ No. %**	**No. tested**	
		**No. %**	**Female**	**Male**			**Female**	**Male**
10–20	0	2 (0.58)	9	6	1 (0.28)	9 (2.58)	9	21
21–30	2 (0.58)	20 (5.72)	56	50	5 (1.43)	44 (12.58)	22	78
31–40	0	33 (9.43)	45	40	5 (1.43)	45 (12.85)	33	79
41–50	1 (0.28)	36 (10.29)	46	39	5 (1.43)	13 (3.72)	9	62
51–60	0	16 (4.58)	15	24	1 (0.28)	13 (9.71)	13	18
61–70	0	6 (1.72)	6	7	0	2 (0.58)	1	4
71–80	0	4 (1.14)	3	4	0	0	0	1
Total	3 (0.85)	117 (33.42)	180	170	17 (4.85)	147 (42)	87	263
Total Together			350				350	

There were no statistically significant differences in any of the case and control groups. However, the patients in middle age groups in control group showed higher rates of IgG+ seroprevalence (*P*=0.002).

According to psychiatric diagnosis based on DSM-IV criteria schizophrenic patients had the highest positive rate (46.28%) and by Chi square test, there is significant difference between IgG and IgM positives in patients and control group; *P*=0.019 and *P*=0.001, respectively. Patients with bipolar mood disorder were the second most prevalent group (20%). Table 2 shows the seropre-valences of *T. gondii* infection in the inpatients according to their psychiatric disorder. The highest prevalence of latent *T. gondii* infection in schizophrenics was found in patients aged 21–30 yr old (Table 3).

**Table 2:** Clinical diagnosis and seroprevalence of anti-*T. gondii* IgG/ IgM antibodies in 350 psychiatric disorders

**Disorder Category**	**Clinical Diagnosis**	**Patients studied**	**Patients with anti-T. gondii IgG+**	**Patients with anti-T. gondii IgM+**
Personality disorders	Personality disorder	6 (1.71)	3 (0.85)	0
Anxiety disorders	Posttraumatic Stress Disorder	15 (4.28)	4 (1.14)	0
Developmental disorders	Mental retardation	6 (1.71)	2 (0.85)	0
Psychotic disorders	Schizoaffective	24 (6.9)	10 (2.85)	1 (0.28)
Psychotic disorders	Schizophrenia	162 (46.28)	65 (18.57)	9 (2.56)
Psychotic disorders	Delusional Disorder	8 (2.28)	4 (1.14)	0
Developmental disorders	Mental disorder	24 (6.85)	8 (2.28)	1 (0.28)
Mood disorders	Bipolar Affective Disorder	70 (20)	33 (9.42)	5 (1.42)
Mood disorders	Major Depressive Disorder	35 (10)	18 (5.13)	1 (0.28)
Total		350 (100)	147 (42)	17 (4.85)

**Table 3:** Seroprevalence of anti-*T. gondii* IgG antibodies in schizophrenic patients according to age groups

**Age groups (yr)**	**Schizophrenic patients**		
	**No. Tested**	**Positive No.**	**%**
10–20	10	2	20
21–30	50	23	46
31–40	56	21	37.5
41–50	31	13	41.3
51–60	12	5	41.6
61–70	3	1	33.3
Total	162	65	40.1

## Discussion

*Toxoplasma gondii* is one of the most widespread protozoan parasites of humans (9), although, prevalence between different populations varies according to different geographical regions. In a previous study on HIV patients in Mashhad, the IgG and IgM anti-*Toxoplasma* antibodies were estimated 38.01% and 2.5%, respectively (21).

In the present study, the prevalence of *T. gondii* infection was 46.85% and 34.28% in psychiatric inpatients and control individuals, respectively. The present results correspond with the result of the other places of Iran (22–26) and some reports in the world (27–31). However, some of them are in contrast (12, 15, 25, 32–35). This difference could be due to our method for selection of population study. Furthermore, the healthy individuals in our control group were the relatives of the patients’ group revealed that the higher prevalence of toxoplasmosis in psychiatric and schizophrenic patients showed associations between *T. gondii* infection and schizophrenia as other researchers have reported (1, 12–14). Patients with schizophrenia have insufficient hygiene and self-care skills, and they have a greater tendency to pica and coprophagia. The prevalence of anti-*T. gondii* IgM antibody was not significantly high in patients in comparison to the control group.

One of the most important aspects of studies on seropositivity of toxoplasmosis in psychiatric patients not be estimated was the initiation of exposure. Indeed, we could not identify either of infection acquired as early as birth time or months/yr later. This is because anti-schizophrenia and bipolar disorder drugs are able to inhibit the growth of *T. gondii* (36).

In addition, environmental exposures have been recognized as a risk to increase the rate of toxoplasmosis. Among behavioral characteristics, known factors associated with *T. gondii* infection such as consumption of untreated water, unwashed raw fruit or vegetable and direct contact with cat played the important role for parasite transmission in our population study.

## Conclusion

The prevalence of *T. gondii* infection in patients with psychiatric disorder especially those suffering from schizophrenia were more in Mashhad compared with control group. Therefore, measuring antibody titer in such patients could be helpful to identify the infection and start treatment in proper time.
